# Head-neck domain of *Arabidopsis *myosin XI, MYA2, fused with GFP produces F-actin patterns that coincide with fast organelle streaming in different plant cells

**DOI:** 10.1186/1471-2229-8-74

**Published:** 2008-07-03

**Authors:** Nadine Walter, Carola L Holweg

**Affiliations:** 1 University of Freiburg, Biology II, Schänzlestrasse 1, 79104 Freiburg, Germany

## Abstract

**Background:**

The cytoskeletal mechanisms that underlie organelle transport in plants are intimately linked to acto-myosin function. This function is mediated by the attachment of myosin heads to F-actin and the binding of cargo to the tails. Acto-myosin also powers vigorous cytoplasmic streaming in plant cells. Class XI myosins exhibit strikingly fast velocities and may have extraordinary roles in cellular motility. Studies of the structural basis of organelle transport have focused on the cargo-binding tails of myosin XI, revealing a close relationship with the transport of peroxisomes, mitochondria, and Golgi-vesicles. Links between myosin heads and F-actin-based motility have been less investigated. To address this function, we performed localization studies using the head-neck domain of AtMYA2, a myosin XI from *Arabidopsis*.

**Results:**

We expressed the GFP-fused head-neck domain of MYA2 in epidermal cells of various plant species and found that it associated with F-actin. By comparison to other markers such as fimbrin and talin, we revealed that the myosin-labeled F-actin was of a lower quality and absent from the fine microfilament arrays at the cell cortex. However, it colocalized with cytoplasmic (transvacuolar) F-actin in areas coinciding with the tracks of fast organelles. This observation correlates well with the proposed function of myosin XI in organelle trafficking. The fact that organelle streaming was reduced in cells expressing the GFP-MYA2-head6IQ indicated that the functionless motor protein inhibits endogenous myosins. Furthermore, co-expression of the GFP-MYA2-head6IQ with other F-actin markers disrupted its attachment to F-actin. In nuclei, the GFP-myosin associated with short bundles of F-actin.

**Conclusion:**

The localization of the head of MYA2 in living plant cells, as investigated here for the first time, suggests a close linkage between this myosin XI and cytoplasmic microfilaments that support the rapid streaming of organelles such as peroxisomes. Potential roles of MYA2 may also exist in the cell nucleus. Whether the low quality of the F-actin-labeling by MYA2-head6IQ compared to other F-actin-binding proteins (ABPs) signifies a weak association of the myosin with actin filaments remains to be proven by other means than *in vivo*. Clues for the mode of contact between the myosin molecules and F-actin so far cannot be drawn from sequence-related data.

## Background

In eukaryotic cells, the acto-myosin system is important for controlling the delivery of diverse cargos [1-3]. Myosin motors exhibit directional stepping along actin filaments, and most of them are capable of binding cellular targets simultaneously. Plant myosins mediate the targeting of diverse organelles, such as the Golgi apparatus [4], the ER [5,6], the mitochondria [7,8], the plastids [9,10], and the nucleus [11]. The acto-myosin system is also involved in the plant cell cycle [12], cell division [13-15], and auxin transport [13,16].

Some characteristics of myosin-related motility may be unique to plant cells. For example, peroxisomal targeting, which requires microtubules in animals, depends on actin microfilaments [17] and myosin [18-20] in plants. Of the three major myosin classes in plants (class VIII, XI and XIII), members of myosin class XI are most often implicated in organelle movement [21,22]. Class XI myosins are broadly distributed in the plant phyla and are represented by 13 isoforms in *Arabidopsis *[23]. They share several features with myosin class V from animals and fungi. One of these features is the extremely long neck, usually consisting of 6 IQ motifs, which may regulate myosin activity by binding to calmodulin or calmodulin-like myosin light chains [22]. According to the lever arm model, the high number of IQs amplifies the motor force of the myosin heads [24,25]. Indeed, biophysical studies of class XI myosins from tobacco and characean alga revealed extraordinary properties: a processive movement along F-actin and strikingly high velocities that are 3–30 times faster than those of muscle myosin II [26-28]. Recent motility assays with an *Arabidopsis *myosin, *AtMYA1*, revealed relatively slow sliding velocities for this class XI myosin [29], showing, however, good agreement with velocities found for cytoplasmic streaming in epidermal cells of *Arabidopsis *[16,30].

The cytoskeletal motility of plant cells is closely linked with the specific cellular architecture [22,31,32]. In general, the cortical F-actin underneath the plasma membrane supports peripheral structures, including the cell wall and the microtubular network. The fine mesh at the cortex protrudes into the region below, i.e. cytoplasm, with long cables of F-actin running through transvacuolar strands. These strands, radiating towards the nucleus and the cell poles, constitute the tracks for rapid cytoplasmic streaming, probably with myosin XI as the main motor. The fine F-actin mesh at the cell cortex probably functions in short distance transport and in co-action with cortical microtubules [33-37], while the longer F-actin strands at the cytoplasmic regions may be linked to vigorous cellular motility. This raises the question of whether these different tasks of F-actin are paralleled by myosins.

Here, we used live cell imaging to study the association of a class XI myosin from *Arabidopsis *with actin filaments in plant cells. We transiently expressed a GFP-fusion protein containing the motor and neck domain of *AtMYA2 *(MYA2-head6IQ) and looked for its localization in various cell-types. The GFP-MYA2-head6IQ produced a different F-actin labeling pattern than standard F-actin-binding proteins. From this observation, and considering the co-distribution of myosin XI with microfilaments used in organelle-transport, we conclude that myosin XI colocalizes with the cytoplasmic F-actin that runs through the transvacuolar strands. Furthermore, we found that the GFP-MYA2-head6IQ, similar to other F-actin-binding proteins, interferes with cellular motility, possibly by blocking endogenous myosin.

## Results

### The head of MYA2 binds to the transvacuolar microfilaments but not to the fine F-actin mesh at the cell cortex

**Figure 1:**
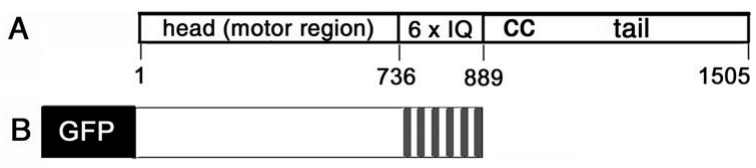
**Diagrams of AtMYA2 and its head-neck domain fused with GFP**. (A) The myosin heavy chain of AtMYA2 (175 kD) consists of the catalytic motor domain containing F-actin-binding capacity, the neck domain with 6 IQ motifs and a consecutive coiled coil (cc) region. The positions of the amino acids are indicated. (B) GFP fusion of the head and neck domain of MYA2 (GFP-MYA2-head6IQ, amino acid residues 1–889). The vector containing GFP alone is not depicted.

**Figure 2:**
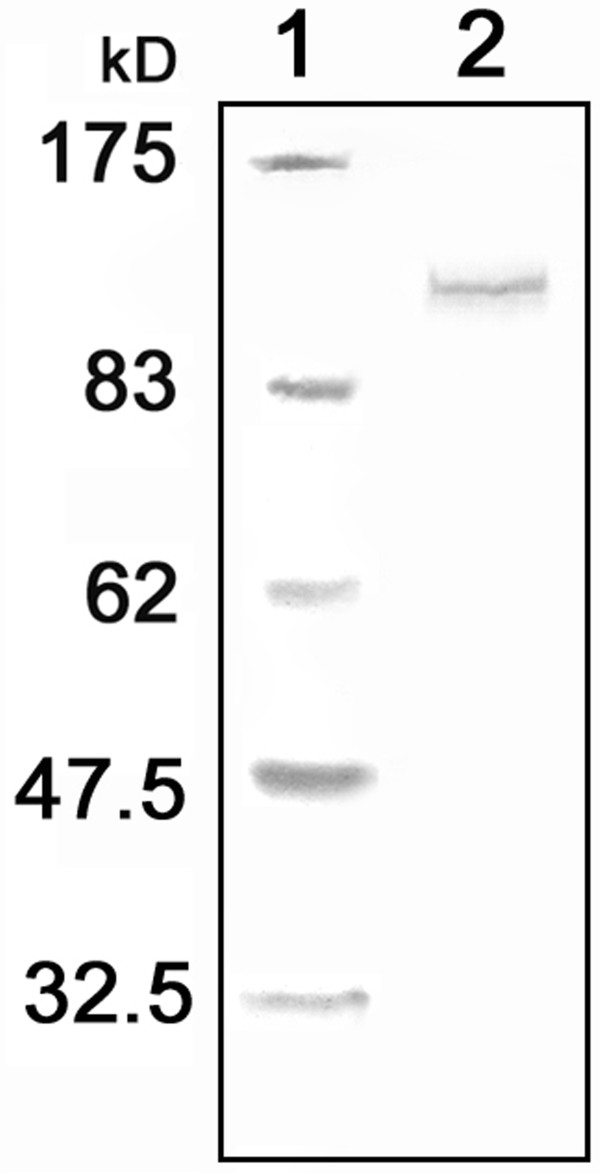
**Verification of GFP-MYA2-head6IQ expression in leaves of *N. benthamiana *by Western blot analysis**. Lane 1 = protein marker for molecular weights. Lane 2 = the analysis of the total protein extract from leaves transformed with GFP-MYA2-head6IQ using anti-GFP antibodies shows the expected size of the protein product (130.5 kD).

**Figure 3:**
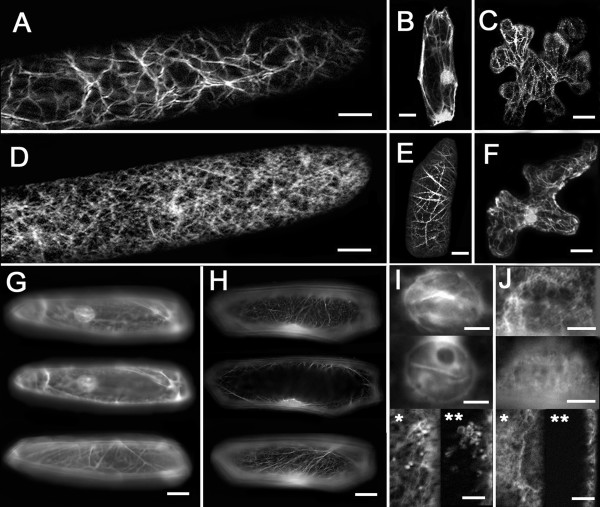
**Localization of GFP-MYA2-head6IQ and F-actin by GFP fluorescence in different plant cells**. (A-C, G, I) F-actin-visualization by GFP-MYA2-head6IQ. For comparison, F-actin is visualized by RFP-FABD2 (D, H, J) and GFP-FABD2 (E) or by YFP-mTn (F). The panels in (G) and (H) show the distribution of F-actin in cells at three different depths. The nuclear localization of GFP-MYA2-head6IQ and RFP-FABD2 signals is shown in panels (I) and (J). Images in (I*, J*) and (I**, J**) derive from optical sections through the center of the cell nucleus and its periphery, respectively. Expression times were 6–7 h for (A) and 15 h for all other images except for 5 d for (C, F). All images represent epidermal cells of *A. cepa *except for (C) and (F) which represent leaf epidermal cells of *N. benthamiana*. Bars in I, J = 5 μm. Bars in all other images = 15 μm.

**Figure 4:**
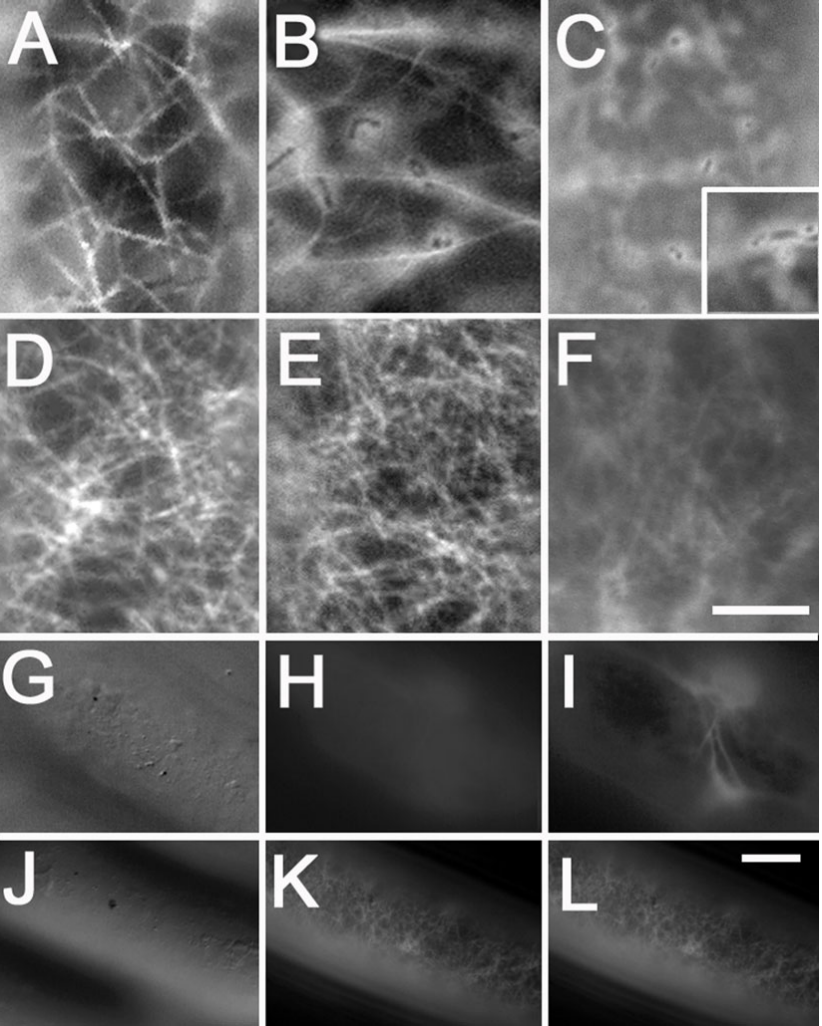
**Sub-cellular localization of GFP-MYA2-headIQ compared to that of RFP-FABD2 in *A. cepa***. F-actin-visualization byGFP-MYA2-head-6IQ at 6–8 h (A), 15 h (B) and 28 h (C) after transformation. The expression of GFP alone results in diffusive cytoplasmic signals (inset in C). F-actin-visualization by RFP-FABD2 at 15 h (D), 25 h (E) and 2 d (F) after transformation. Spatial differences in the F-actin net either labelled with GFP-MYA2-head6IQ (G-I) or RFP-FABD2 (J-L). The focus planes of the upper cell surface (gold particles are visible as black dots) shown by DIC images in (G) and (J) compared to the corresponding fluorescent images in (H) and (K). Images in (G, H) and (J-L) have been acquired from the same plane while the image in (I) derives from a plane 5 μm below. Bar for images in (A-F) in (F) = 5 μm, for images in (G-L) in (L) = 10 μm.

**Figure 5:**
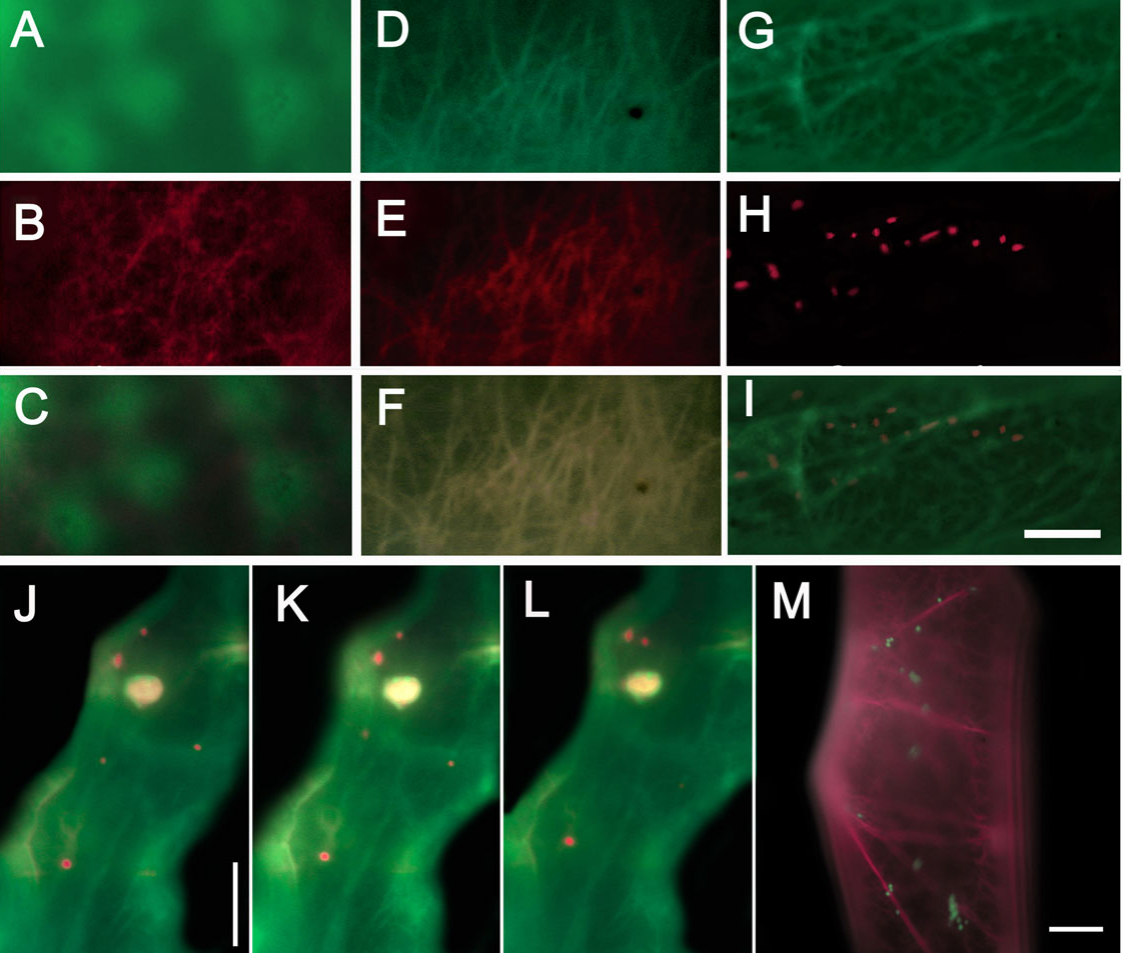
**F-actin-binding of GFP-MYA2-head6IQ and RFP-FABD2 in respect to microfilament subpopulations and peroxisome pathways in different plant cells**. (A-I) Co-expression of fusion proteins in *A. cepa: *GFP-MYA2-head6IQ (A) with RFP-FABD2 (B), YFP-mTn (D) with RFP-FABD2 (E) and GFP-MYA2-head6IQ (G) with a peroxisomal marker fused with mcherry (H). Merged images are depicted in (C), (F) and (I), respectively. (J-L) Two-sec-interval images of an epidermal cell of *Sinapis *cotyledons co-transformed with GFP-MYA2-head6IQ (GFP in green) and a peroxisomal marker (mcherry in red). (M) Co-transformation of a cell of *A. cepa *with RFP-FABD2 (RFP in red) and a peroxisomal marker (CFP in green). Widefield fluorescence images were taken at 15 h (A-I) or 18 h (J-M) after transformation. Bar in (I) = 5 μm for all images in the upper panel. Bars in (J-M) = 10 μm.

The motor head and 6 IQs from AtMYA2 (Fig. 1A) were introduced into the plant expression vectors pCAT-GFP and pBIN. The expression of the fusion protein, GFP-MYA2-head6IQ (Fig. 1B), in the cells of various plant species, resulted in the labeling of the actin filaments. Western blot analysis proved that the MYA2-GFP-head6IQ was fully translated (Fig. 2). In the epidermal cells of *Allium cepa *(Fig. 3A, B), *Nicotiana benthamiana *(Fig. 3C and Fig. 4A), or *Sinapis alba *(Fig. 5J–L), F-actin decoration by this construct revealed a filamentous network extending towards the cell cortex with cables running through longitudinally and nucleus-oriented cytoplasmic strands.

This pattern was consistently displayed in all of the cells examined, without differences in the density of the microfilament network; it was independent of the expression level. Since the net produced by GFP-MYA2-head6IQ applied to a typical F-actin organization, we tested for possible differences from other F-actin-binding proteins. We used GFP-talin (mTn) and RFP- or GFP-FABD2, which contains the actin-binding domain 2 of *Arabidopsis *fimbrin 1 known to produce the best quality of F-actin imaging in living plant cells [38-42]. For comparison, we introduced these fluorescently coupled ABPs into the same plant tissues (Fig. 3D–F).

Because of the strong expression due to the double 35S-promoter, a fluorescent signal from the head-neck-fusion appeared very shortly after transformation (6–8 h) and was clearly associated with F-actin (Fig. 3A). However, the imaging of F-actin revealed that the GFP-mTn- and RFP- or GFP-FABD2-labelled networks were much denser than the relatively loose mesh visualized by GFP-MYA2-head6IQ (compare Fig. 3A–C, G with 3D–F, H and Fig. 4A, B with 4D, E). Cells expressing the myosin head fusion protein showed a filamentous pattern with fewer cross-links and a slightly higher cytoplasmic background. After longer expression times, the GFP-MYA2-head6IQ signal became increasingly cytoplasmic and accumulated at diverse cellular sites (Fig. 3B and Fig. 4B). In addition, the GFP-MYA2-head6IQ produced a less constricted signal (Fig. 3G) than the RFP-FABD2 signal (Fig. 3H), which sharply outlined the filaments. Thus, the myosin-decorated microfilaments often appeared slightly wider compared to those of other ABPs (compare Fig. 3A with 3B, Figs. 4A, B with 4D, E and Figs. 5G with 5C and 5F). At more than one day after transfection, the GFP-MYA2-head6IQ molecules were localized almost entirely in the cytoplasm (Fig. 4C), while the F-actin labeled by RFP-FABD2 remained unchanged for at least 25 h (Fig. 4D, E), and only disappeared at 2 d after transformation due to the normal decay in cellular viability (Fig. 4F).

The F-actin reporter RFP-FABD2 also produced different filamentous patterns near nuclei. The GFP-MYA2-head6IQ marked highly fluorescent short bundles in nuclei (Fig. 3I) or, occasionally, fluorescent patches in or close to nuclei (Fig. 3I*/I**); these were absent in cells expressing RFP-FABD2 (Fig. 3J).

Generally, RFP- and GFP-FABD2-labeled actin filaments appeared at various cellular sites: as microfilaments extending from the cell cortex towards the plasma membrane and as microfilaments running through cytoplasmic strands (Fig. 3E, see also Fig. 5M). Microfilaments labeled by GFP-MYA2-head6IQ were found only in the more internal regions of cells. This was noted when the plane of the F-actin net was focused under the microscope, starting from the uppermost surface of cells (Fig. 4G, I, K, L). While comparing cells expressing either GFP-MYA2-head6IQ or RFP-FABD2, it became evident that the plane of focus of the FABD2-labelled F-actin net was nearly identical to that of the cell surface (Fig. 4K, L). In contrast, the plane of the main focus of myosin-labelled microfilaments never appeared in these cortical regions (Fig. 4G, H), but localized in regions more than 5 μm below the cell surface (Fig. 4I).

### GFP-MYA2-head6IQ dissociates rapidly and is not able to compete with other ABPs

To detect further differences in the labeling of F-actin, GFP-MYA2-head6IQ and RFP-FABD2 were simultaneously introduced into cells (Fig. 5A–C). The actin filaments were exclusively decorated by the fimbrin protein (Fig. 5C), while the myosin was localized entirely in the cytoplasm (Fig. 5A). To test whether other ABPs respond in a similar manner, due to possible competition for binding sites or limited space along F-actin, RFP-FABD2 was co-expressed with YFP-mTn. As result, a similar actin network was visualized, with fully overlapping signals (Fig. 5D–F). None of the ABPs displayed enhanced cytoplasmic localization. When YFP-mTn was co-expressed with GFP-MYA2-head6IQ, the myosin was again detached from filamentous actin (data not shown). However, if the GFP-MYA2-head6IQ was co-expressed with a peroxisomal marker under similar conditions, its labelling ability was preserved (Fig. 5G–I).

### The overexpression of GFP-MYA2-head6IQ interferes with fast organelle motility

We then questioned whether the MYA2-decorated actin filaments reflect the main pathways of cytoplasmic movements. To address this, we focused on the cytoplasmic strands with the most vigorous streaming of organelles and compared their distribution with the GFP-MYA2-head6IQ signal distribution. We also examined peroxisome targeting in cells by co-bombardment of a peroxisomal marker and the GFP-MYA2-head6IQ or RFP-FABD2 fusions (Fig. 5J–M). The most vigorous movement of organelles was observed in longitudinal and nucleus-oriented strands that, along with the actin cables, occupied the more internal (cytoplasmic) regions of the cell. Thus, peroxisome highways appeared to coincide with the thicker, more longitudinal filaments labeled by the GFP-MYA2-head6IQ (Fig. 5J–L) or RFP-FABD2 (Fig. 5M). The apparent elongated shape of a peroxisome that co-distributes with the myosin-labeled strand in the center of Fig. 5H indicates that the organelle was moving rapidly during imaging.

**Figure 6:**
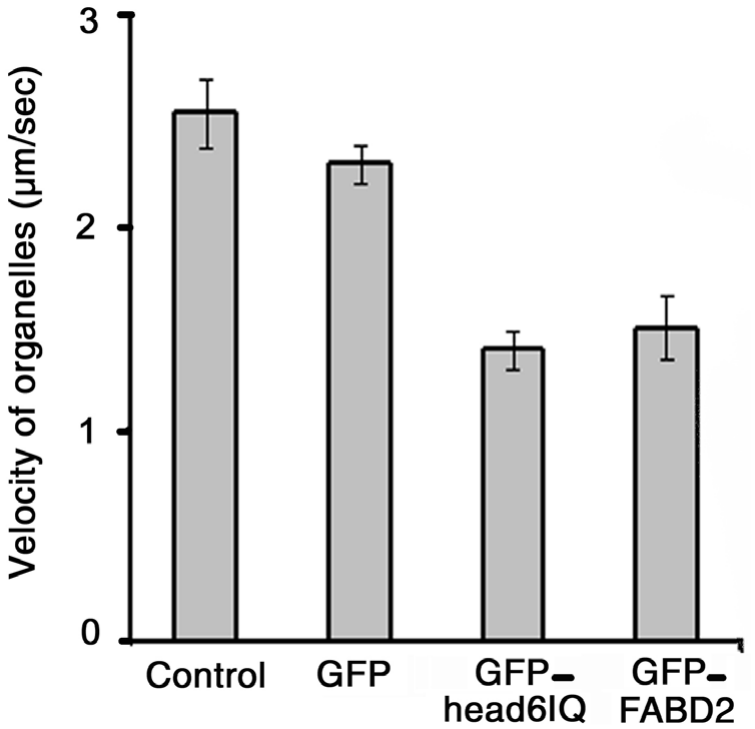
**Comparison of streaming velocities in epidermal cells of *A. cepa***. Maximal streaming velocity of particles in untransformed cells (control), in cells expressing GFP (GFP), the vector containing the GFP-fused head-neck of MYA2 (head6IQ) or the vector containing the GFP-fused fimbrin actin-binding domain (FABD2). Error bars, SE (n = 12–16).

Because the transport of organelles is mediated by myosin, overexpression of other F-actin-binding proteins is expected to interfere with cytoplasmic streaming by blocking the attachment of myosin to actin filaments. As shown previously, such dominant negative effects on cytoplasmic streaming exist for GFP-mTn and GFP-FABD2 overexpression [16]. In order to examine the possibility that excess GFP-MYA2-head6IQ would interfere with the binding of endogenous myosin's to F-actin, we transformed cells of *A. cepa *and measured the maximal streaming velocities with time-lapse images. Focusing only on rapidly moving particles to obtain "mean maximal speeds" has been proven to be a suitable approach for evaluating changes in cytoplasmic streaming [16]. In untransformed cells, the mean maximal speed was 2.5 μm/sec; the expression of GFP-MYA2-head6IQ and GFP-FABD2 reduced this by about 40% without significant differences between the ABPs (Fig. 6). In control cells expressing GFP alone, the mean maximal speed was significantly decreased by about 10% (calculated at the 99% level of significance). Thus, it appears reasonable to assume that the net reduction of the maximal streaming velocity caused by myosin or fimbrin overexpression was about 30%.

### Comparison of potential actin-binding regions in myosin head domains

**Figure 7:**
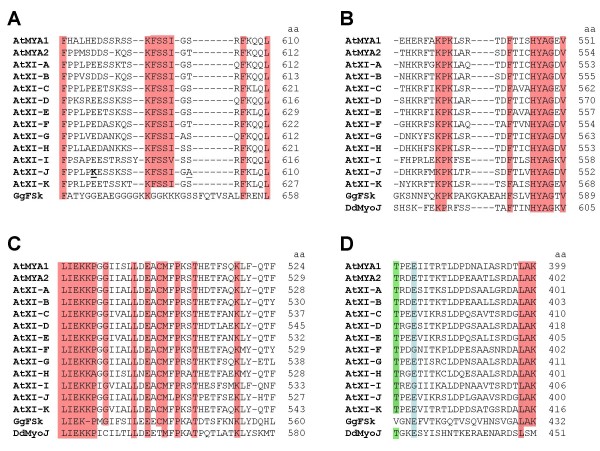
**Comparison of several potential actin-binding sites and the TEDS site on myosin heads**. Alignment of deduced amino acid sequences of myosin class XI of *Arabidopsis*, DdMyoJ and muscle myosin GgFSk. (A) Surface loop 2 involved in the binding of the first actin monomer. (B) Region suggested for binding the second actin. (C) Region that mediates the major contact during primary actin-binding. (D) TEDS rule site with glutamic acid (blue shaded) and, close to it, threonine (green shaded). At *Arabidopsis thaliana*, Dd *Dictyostelium discoideum*, Gg *Gallus gallus*. Conserved amino acids are red shaded. DdMyoJ was excluded from the alignment in (A). Surface loops according to Schröder et al. [45]. In each sequence, the position of the last amino acid residue is indicated (aa).

Structural analysis of the motor domain of myosins has revealed that various regions and surface loops are involved in the contact between actin and myosin [43-45]. To find possible clues to the interaction of MYA2-head6IQ with F-actin, we aligned the amino acid sequences of several loops and the TEDS rule site [46] from all class XI myosins in *Arabidopsis*, from *Dictyostelium *myosin (DdMyoJ), and from skeletal muscle myosin (GgFSk) (Fig. 7). The first actin binding site is partially conferred by loop 2 (Fig. 7A). In all sequences, it bears one or two negatively and 4–6 positively charged amino acids. Thus, even though the loop 2 sequences are quite divergent, they have similar ionic strengths.

Exceptionally, AtXI-J has additional alterations in charge and polarity not present in the other sequences (residues underlined). A net positive charge also prevails in the secondary actin-binding site (Fig. 7B). In all sequences, a total of 15–16 hydrophobic amino acid residues are in a conserved region which contributes to the primary actin-binding (Fig. 7C). At the TEDS rule site, which is named on the basis of these amino acids, regulates motor activity by phosphorylation in some myosins [46], all sequences have a unphosphorylatable and negatively charged glutamic acid (Fig. 7D). However, a phosphorylatable threonine residue is proximal to the TEDS site in DdMyoJ and all plant myosins.

## Discussion

The biology of organelle streaming, in terms of myosin traveling along an F-actin filament, is enigmatic. Although recent experiments with myosin tails have greatly contributed to our understanding of the target-related functions of plant myosins [18-20,47], the interaction of plant myosins with F-actin is still not completely understood.

Since myosins were identified as actin filament-based biochemical machines, a huge number of *in vitro *studies have identified their biophysical properties including velocity, power, and F-actin-binding affinity [48,49]. Studying the interaction between plant myosins and F-actin has remained limited, perhaps, due to the difficulties in obtaining protein from tissue or heterologous expression systems. Nevertheless, in order to study the interaction between *Arabidopsis *myosin MYA2 and actin filaments, we applied *in vivo *microscopy combined with transient expression assays. This approach took advantage of the complexity that occurs in live cells, including the presence of cytoskeletal interactors and factors that may be involved in acto-myosin function. The use of the previously described *mya2 *mutant [30] was not possible due to a second deletion which has since been detected [50]. Despite the success of GFP-MYA2-head6IQ expression in all plant species, we could not identify expressing cells of *Arabidopsis *with a quality suitable for F-actin imaging. The small size of the cells and the diffusive tendency of the protein, together with problems of plant transformation, hindered our plan to maintain a homologous system.

### GFP-MYA2-head6IQ signals produce a pattern coinciding with long cytoplasmic F-actin strands

As shown here, the head-neck of MYA2 fused to GFP is functional and can recognize filamentous actin. In epidermal cells, it preferentially labeled microfilaments in cytoplasmic regions, i.e. in transvacuolar strands, and was rarely found in cortical F-actin. While other ABPs, such as GFP-FABD2 or YFP-mTn, associated more ubiquitously with F-actin, the GFP-MYA2-head6IQ was absent from the fine arrays at the cell cortex. The observation that the myosin head often paralleled pathways of fast streaming organelles suggests its association with F-actin populations specific to long-distance transport.

The question arises as to how this specificity is determined. Actin assembly and remodeling is regulated by a variety of ABPs that recognize actin pools or control actin stability [39,51-53]. In pollen tubes, for instance, different actin polymer-binding domains fused to GFP label different populations of F-actin [54]. Perhaps myosins distinguish between actin polymers based on the composition or dynamics of actin monomers, or with the help of other F-actin- or myosin-binding proteins. For instance, a less dynamic actin net could exist in cytoplasmic strands that may be recognized by myosins that support long-distance transport. Myosin heavy chains present various possibilities for protein-protein interactions. Repulsive as well as attractive forces produced by side chains, ion strength, phosphorylation, or the association with other proteins can positively or negatively influence the binding and motility of myosin motors [see respective chapters in ref. [49]]. The ability to associate with filamentous actin can even be determined by the myosin tail [2,55]. In the case of MYA2, the tail itself lacks F-actin-binding capacity [19,20].

According to motif recognition programs [56,57], the head subdomains of MYA2 include F-actin- and ATP-binding sites and sites for phosphorylation. Other motifs exist with yet unknown function, such as Myosin N or SH3-like domains (amino acid residues 10–53), MurB (662–716), and FARP (207–217). The neck domain (735–889), following the motor core, contains 6 IQ motifs believed to bind myosin light chain binding, probably calmodulin [58]. A tropomyosin-like domain, which enhances F-actin-binding in conventional myosin II, spans from amino acids 851–1058. Based on this data it is not possible to predict the association of MYA2 with actin filaments more precisely. F-actin affinity studies with other ABPs, such as FABD2, revealed a stoichiometry of 1:4 [40] and ADF-mediated F-actin turnover was inhibited in the presence of mTn [59]. Due to the difficulties of *in vitro *protein production, such assays were not performed for MYA2. In addition, we cannot draw conclusions about the natural situation of ABPs. Dimerization of two head molecules via the coiled-coil domain, as proposed for other members of class XI [28,60], was not expected because this domain was not included in our construct.

### Potential roles of MYA2 in the cell nucleus

The increase of fluorescent signal in the nucleus following overexpression of ABPs appeared to be specific for MYA2. The fact that other ABPs, such as GFP-mTn [61] and GFP- or RFP-FABD2, never produced a similar accumulation cannot be explained by the different molecular weights, because myosin is much larger (130.5 kD versus less than 70 kD for other ABPs). The GFP-MYA2-head6IQ signals produced conspicuous rod-like formations in or at the nucleus, reminiscent of the actin bundles that were observed in grooves and invaginations of tobacco nuclei [62]. Considering these findings and other observations showing myosin I-dependent nuclear transport of RNA polymerase II [63], our observations suggest that MYA2 may function in mRNA export. Whether MYA2 has roles in the cell cycle and cell division requires further analysis.

### The localization of GFP-MYA2-head6IQ suggests a weak in vivo F-actin-binding

The lower quality of filamentous signals induced by the GFP-MYA2-head6IQ, compared with other F-actin markers, is not fully explained by artifacts due to overexpression or protein degradation. The expression times for the transient assays were short and the protein expressed in leaves of *N. benthamiana *was still functional more than 5 d after transformation. Secondly, even though the amount of the GFP-MYA2-head6IQ protein greatly exceeded that of RFP-FABD2 during the co-expression experiments, the cells still displayed a relatively normal RFP-FABD2-actin network. Despite some minor declines in the quality of the cortical F-actin, we detected neither microfilament bundling nor depolymerisation (Fig. 5C). Moreover, the quality of this net is similar to those in cells that expressed GFP alone (data not shown). GFP is known to produce cytoplasmic signals that do not severely affect F-actin function (compare also Fig. 6).

Nevertheless, the cytoplasmic signal patches observed in all GFP-MYA2-head6IQ expressing cells suggest that a considerable amount of molecules were not bound to actin filaments. This circumstance cannot be explained by a low number of binding sites on F-actin because myosin binds frequently to it [28,49] and microfilament decoration was rich. Even though the patches could indicate some disruption of the cytoplasmic actin strands, this subpopulation of long filaments was still preserved and able to sustain organelle trafficking quite similarly to GFP-FABD2 (see Fig. 6).

Another explanation for the diffusive behavior of the myosin may be found in the inability of the head to compete with FABD2 or mTn. This inability might point to different mechanisms for F-actin-binding, which are possibly weaker for the myosin head. Recent *in vitro *motility assays, with a recombinant head of AtMYA1, revealed a high ratio of F-actin-bound states [29]. This suggests a tight contact with F-actin, *in vivo*.

However, the modular arrangement of ABPs as well as the potential co-action with other factors *in vivo *might influence F-actin binding quite differently. In a study of FP-fusions of *Arabidopsis *fimbrin 1 [64,65], F-actin-binding varied from a more distinct network to enhanced cytoplasmic localization, dependent on the sequence length used for the different constructs. Therefore, the observed weak association of the GFP-MYA2-head6IQ with F-actin might be a consequence of the conditions *in vivo *or related to intrinsic features of the MYA2 protein.

Multiple points on myosins may coordinate actin filament association including surface loops and positive or negative charges. Ideas about these contacts came from high-resolution crystallography of conventional myosin [44,45]. Several similarities and conserved amino acids exist in myosin surface loops between different phyla. Our comparison of the TEDS site and surface loops in myosins from chicken muscle, *Dictyostelium *and *Arabidopsis *also revealed conservation of important amino acid residues suggesting consistency in ionic strength and hydrophobicity. The high degree of functional conservation between the actin-binding residues in myosins of plants and other phyla might indicate a similar contact with F-actin. Whether variations in individual sequences could affect this contact such as charge and polarity changes in loop 2 of AtXI-J or the phosphorylatable threonine residue in class XI myosins [23] and in DdMyoJ can not be predicted. Analysis of the kinetics and mechanical properties of plant myosins might be a key for enhanced understanding the mode of F-actin-binding. Dissecting the modular organization of myosin by head truncations or mutations of amino acid residues might also help to resolve this question.

### GFP-MYA2-head6IQ affects cytoplasmic streaming in a similar manner to other ABPs

In a former study, it was shown that the method of determining the maximal streaming velocity by the selection of only three of the fastest particles in a cell results in reliable values and small standard deviations [16]. In good accordance with these results including the velocity reduction following ABP-overexpression in epidermal cells of *Arabidopsis*, we observed that the maximal velocity was reduced by GFP-FABD2 by at least 30% in cells of *A. cepa*. The expression of GFP-MYA2-head6IQ had the same effect. In the light of competition experiments, in which the head of MYA2 appeared to be displaced by FABD2, a more severe reduction of streaming might have been expected for the myosin. Interestingly, other authors reported that overexpression of the tail of myosin AtXI-K nearly halted organelle motility [47]. That the negative effects caused by the displacement of endogenous myosins by GFP-MYA2-head6IQ molecules are comparable low may be explained by different scenarios.

First, a variety of myosin isoforms is usually present in a cell. Numerous reports demonstrate that *Arabidopsis *myosins of class XI share common functions, such as the transport of peroxisomes [18-20,47]. However, negative effects resulting from overexpression of their tails or RNAi were shown to be highly variable among different isoforms [47]. Compared to XI-K, MYA2-induced effects were only moderate.

Secondly, the well preserved streaming could again be a result of the complex structure that endows endogenous myosins with much better F-actin-binding ability. The double-head formation of myosin XI is known to be required for processive movement along F-actin [28] and indispensable for fast organelle transport [29]. If dimerisation of endogenous myosins enhances F-actin-binding, which itself activates myosin ATPases [48,66], quite different myosin binding and sliding dynamics might be expected. Finally, other subdomains, such as the tail [2,55], could influence the acto-myosin complex and thereby protect endogenous myosin from disturbances by GFP-MYA2-head6IQ or other GFP-fused ABPs.

## Conclusion

Our study of MYA2 in live plant cells by overexpression of its head-neck domain suggests that this myosin XI associates predominantly with cytoplasmic F-actin involved in the rapid movement of organelles. Roles of MYA2 may also exist in the cell nucleus. The comparison of potential actin-binding sites on myosins between different phyla did not allow making assumptions about different contacts between F-actin and myosin isoforms from *Arabidopsis*. So far, under conditions in live cells, this contact seems to be weak in case of the MYA2 head-neck either indicating a flexible binding mode or that other factors might be involved such as the myosin tail or other cytoskeletal proteins.

## Methods

### Constructs

The coding region of *AtMYA2 *[TAIR:At5G43900] [66] (Fig. 1A) was amplified from cDNA obtained from Arabidopsis (Columbia) leaves via RT-PCR. Using the GFP-FABD2 vector [41], a derivative of pCAT-GFPm3 [68], we prepared an N-terminal GFP-fusion with the motor head of *AtMYA2 *including the neck domain (MYA2-head6IQ, amino acid residues 1–889, Fig. 1B) for transient expression of proteins. The PCR primers used for the MYA2-head6IQ construct were 5'-ATAGATCTTATGGTTGCTAACTTCAATCCAT as the forward primer and 5'- ATACTAGTTTGGCTGCTTGGAGTGCTCCAGTTTCTCTA as the reverse primer. The PCR products were cut with the respective enzymes *Bgl*II and *Spe*I (as underlined) and ligated into the pCAT-GFPm3 vector via the *Bam*HI and *Spe*I sites. The same vector containing the 35S promoter was also used for expression of GFP alone. For protein expression in cells of *N. benthamiana*, the expression cassette of pCAT-GFPm3 containing GFP-MYA2-head6IQ was cut with *Hind*III and the fragment of 4.6 kb was ligated with the binary expression vector pBIN20 also cut with *Hind*III. The recombinant genes were verified by sequencing. In addition to a pCAT construct containing GFP alone, we used GFP-FABD2 [41] and RFP-FABD2 (a kind gift of P. Nick, unpublished; cloned according to RFP-Arp3 into the transient 35S-p2RGW7 Gateway vector from Invitrogen) and a vector containing a 35S-YFP-mTn expression cassette [69] for the visualization of F-actin. For peroxisomal localization, we used RFP and CFP fusions with the type 1 peroxisome targeting signal [19].

### Transformation of plants

In case of transformation of *Allium cepa*, pieces were cut from onion bulb epidermis under sterile conditions and placed on 6 cm Petri dishes containing half-strength MURASHIGE and SKOOG salts with vitamins (DUCHEFA), 0.1% MES, 1% (w/v) sucrose and 1.5% (w/v) agar-agar (ROTH). In case of *Sinapis alba*, cotyledons of 10 d old plants were transformed abaxially. Epidermal cells were bombarded with gold particles that had been coated with DNA as previously described [70]. After transformation, Petri dishes were placed for 5–30 h at 23°C in the dark. Visualization of GFP-conjugated myosin heads was usually done after 6–8 h expression. Longer expression times were used for various purposes, such as analysis of protein dissociation or competition assays.

For infiltration of tobacco leaves with Agrobacteria the abaxial surface of *Nicotiana benthamiana *leaves was infiltrated by Agrobacterium tumefaciens harboring the binary plasmid vector pBIN20 which contained the expression cassette of pCAT-GFP or pCAT-GFP-MYA2-head6IQ. Higher peaks of protein expression were achieved by the simultaneous infiltration of 35S-P19 in pBin61 [71] proven to reduce gene silencing after infection with Agrobacteria. Images from leaf epidermis were taken after 5 d growth at 23°C, at the peak of protein expression with more than 70% of epidermal cells showing fluorescence.

### Biochemical determination of head protein expressed in leaves of N. benthamiana

Leaves were ground in liquid nitrogen and protein extracts from about 200 mg leaf powder were prepared by boiling for 5 min at a 1:1.5 (w/v) ratio in the following SDS-buffer: 65 mM Tris/HCl pH 7.5, 4 M urea, 3–5% SDS, 10% beta-mercaptoethanol, 15% glycerol and 0.05% bromphenol blue. Aliquots of 25 μl protein extract for GFP-MYA2-head6IQ and 10 μl for GFP alone were subjected to SDS-PAGE followed by electroblotting onto nitrocellulose membranes and immunoblot analysis. Primary anti-GFP antibody (from rabbit; Acris) was used for detection of the GFP-fusion and anti-rabbit alkaline phosphatase-conjugated antibody (Calbiochem) was used as secondary antibody. A broad range (6–175 kD) prestained protein marker (New England Biolabs) was used for the estimation of molecular weights of proteins. Development of the blots was carried out with a solution of nitroblue tetrazolium/5-bromo-4-chloro-3-indolyl-phosphate (Roche).

### Microscopy

Live cell and fluorescence imaging was performed on a Zeiss (Jena, Germany) AxioImager Z1 equipped with a Zeiss AxioCam and Axio Vision Rel. 4.5 software. GFP fluorescence was observed with excitation at 470 nm and emission at 525 nm, CFP with 436/480 nm. RFP and mcherry were recorded through the filter set 560/645 nm. In order to avoid bleed-through of fluorescence during co-expression of YFP-mTn and RFP-FABD2, YFP was recorded through the GFP filter set. In addition, the limit for exposure to the RFP excitation was tested in a cell that expressed YFP-mTn alone (the limit was 1.0 sec). During co-expression experiments, RFP was excited for shorter times. Due to the diffusive tendency of the GFP-MYA2-head6IQ, exposure times between 300 and 700 msec were used for widefield fluorescence microscopy proving to be a fast and appropriate method for studying its distribution.

Images were processed with Adobe Photoshop 5.5. Images in Fig. 3(A,D) represent 3D projections of 20 images from optical sections taken with the 63 × objective at 1 μm increments by the Apotome function of the microscope. Fifteen cells were observed in this manner. Images in Fig. 3(B,C,E,F) are 3D projections of images resulting from optical sections made by laser scanning microscopy (Zeiss LSM 510) taken with the 63 × (B, E) and the 40 × (C, F) objective (n = 30 cells). For Fig. 3(B,E), 25 optical sections were taken for each cell at 1 μm increments. For Fig. 3(C,F) about 15 optical sections were taken at 2 μm increments. The images in Fig. 3(G,H,I,J) derive from widefield fluorescence microscopy, except for (I*, J*) and (I**, J**), which represent two optical sections through the nuclear region taken by the Apotome function. Images in Figs. 4 and 5 were made by conventional (widefield) fluorescence microscopy. The number of observed cells was 15 for each image in Fig. 4(A–F), 10 for 4(G–L) and n = 5 for each image in Fig. 5.

In order to determine the spatial differences between the nets of F-actin that were labelled by the different ABPs, the uppermost surface of 10 cells for each construct was focussed upon with DIC optics with the 40 × or the 63 × objective. Then, stepping slowly through the z-axis under the respective excitation wavelength, the μm display was observed until the F-actin net became clearly visible.

For the determination of maximal streaming velocities of organelles cells were observed with a 63 × plan apochromat oil immersion system N.A 1.4 objective supplied with DIC optics. We focused on all kinds of particles without specifying organelles. Velocities of 40–50 particles from 12–16 cells (= n) for each construct were determined using 30 time-series images taken at 1 sec intervals and analysed frame by frame with Image J (National Institutes of Health, Bethesda). As the velocity range of particles in a single cell is very broad, the selection of three of the fastest particles per cell proved to be a fast and direct method which produced low standard deviations. Monitored by the scroll function in Image J these particles were followed for as long as fast translocation proceeded (5–20 μm). The distance in μm covered by a particle between the first and the last image was determined by the "analyze-measure" function of IMAGE J and divided by the respective number of images, i.e. seconds. To determine the statistical significance of potential differences in organelle velocity, the data were analysed by a one-way analysis of variance (ANOVA) followed by a Student's *t*-test at 90% and 99% significance levels. Standard errors were calculated for error bars (Fig. 6).

### Sequence alignment

Sequences of *Arabidopsis *myosins were obtained from TAIR [72], for AtMYA1 [AT1G17580], AtMYA2 [AT5G43900], AtXI-A [AT1G04600], AtXI-B [AT1G04160], AtXI-C [AT1G08730], AtXI-D [AT2G33240], AtXI-E [AT1G54560], AtXI-F [AT2G31900], AtXI-G [AT2G20290], AtXI-H [AT4G28710], AtXI-I [AT4G33200], AtXI-J [AT3G58160] and AtXI-K [AT5G20490]. The sequence of *Dictyostelium *myosin DdMyoJ was obtained from [SwissProt:.P54697], that of chicken muscle myosin GgFSk from [SwissProt:P13538]. The names are as in the tree of Hodge and Cope [73]. Amino acid residues of surface loops were aligned in input sequence using CLUSTAL W (version 2.0.2) [74]. A third loop involved in the primary actin binding [45] was not included. Because of too low similarity, DdMyoJ was excluded from Fig. 7A.

## Authors' contributions

NW carried out the cloning, biolistic transformation and parts of the microscopical work. CLH carried out the conception of the work, made biochemical and microscopical studies, and wrote the manuscript.
